# Supplementary material for: Complex reaction processes in combustion unraveled by neural network-based molecular dynamics simulation
Source: Nat Commun. 2020 Nov 11;11:5713. doi: 10.1038/s41467-020-19497-z (PMC7658983; doi:10.1038/s41467-020-19497-z)
Supplement: Supplementary file 1 — Supplementary Information [file 41467_2020_19497_MOESM1_ESM.pdf]

Supplementary Materials

Complex Reaction Processes in Combustion Unraveled by Neural Network Based Molecular Dynamics Simulation

Jinzhe Zeng[1], Liqun Cao[1], Mingyuan Xu[1], Tong Zhu[1,2,]* and John ZH Zhang[1,2,3]*

[1]Shanghai Engineering Research Center of Molecular Therapeutics & New Drug Development, School of Chemistry and Molecular Engineering, East China Normal University, Shanghai, 200062, China

[2]NYU-ECNU Center for Computational Chemistry at NYU Shanghai, Shanghai, 200062, China

[3]Department of Chemistry, New York University, New York 10003, United States

Table of Contents

- The performance of the NN model on the prediction of energy and atomic forces (Figure 1 and Table 1).
- Time dependences of the total energy and temperature during the MD simulation under NVE ensemble (Figure 2).
- Time dependences of the numbers of main radicals created during the MD simulation (Figure 3).
- Time dependences of the potential energy of the system during the MD simulation (Figure 4).
- Real-time trajectory of the formation of cyclopropene (Figure 5).
- Rate constants of several important reactions predicted from the trajectory (Table 2).
- Supplementary Note
  List of reactions found in the trajectory

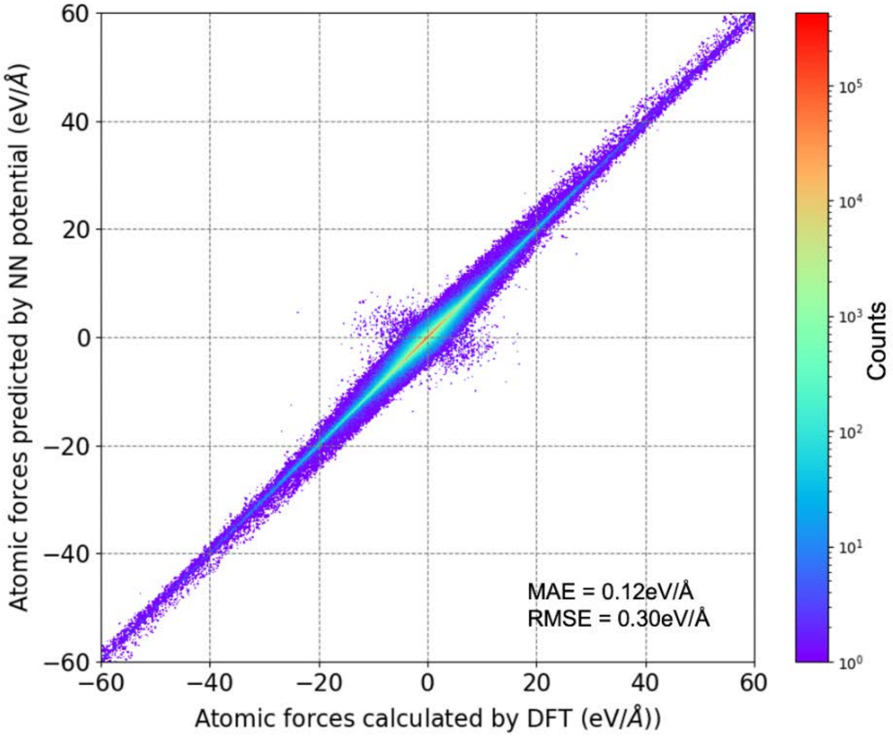

Figure 1. The correlation of atomic forces between NN predictions and DFT calculations. There are still a few points in the area with an absolute value greater than 60eV/Å. To make the picture clearer, they are not shown. However, these points were included when calculating the MAE and RMSE values. Considering such a large value range, the accuracy of the NN model is encouraging.

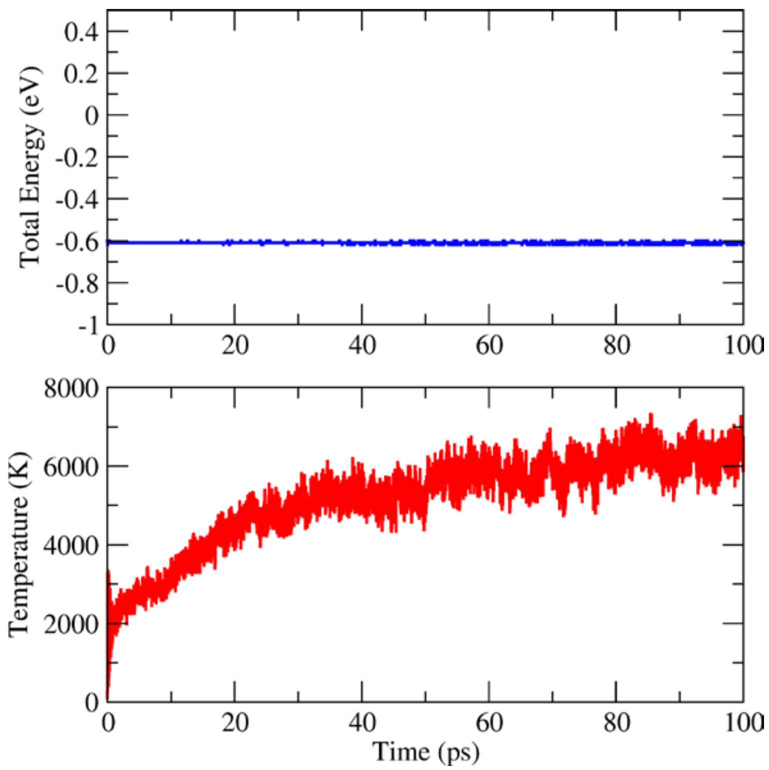

Figure 2. Time dependences of the total energy (the relative value to the first snapshot) and temperature during the MD simulation under NVE ensemble.

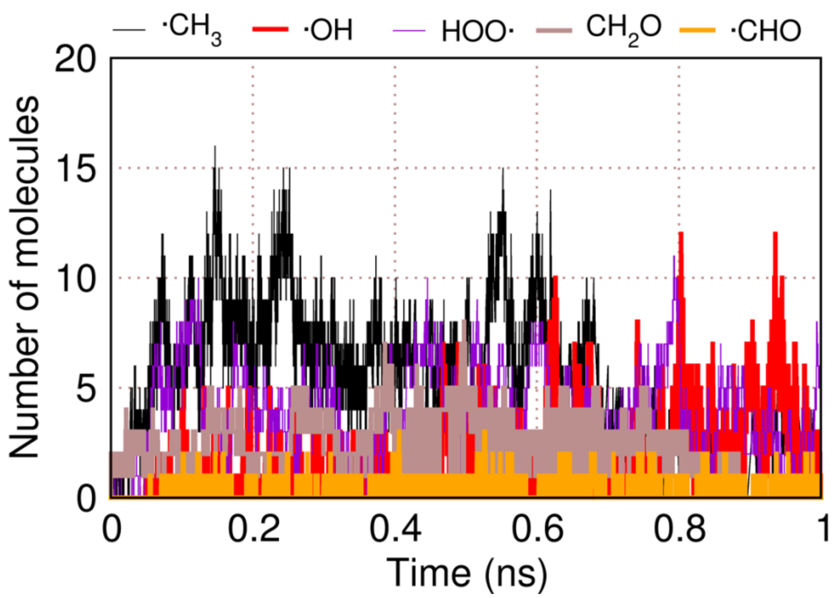

Figure 3. Time dependences of the numbers of main radicals created during the MD simulation.

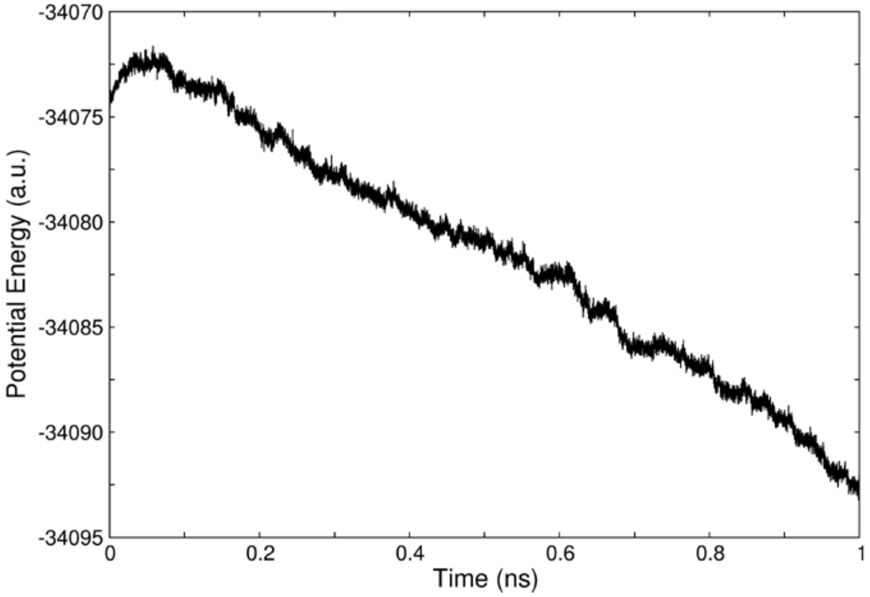

Figure 4. Time dependences of the potential energy of the system during the MD simulation.

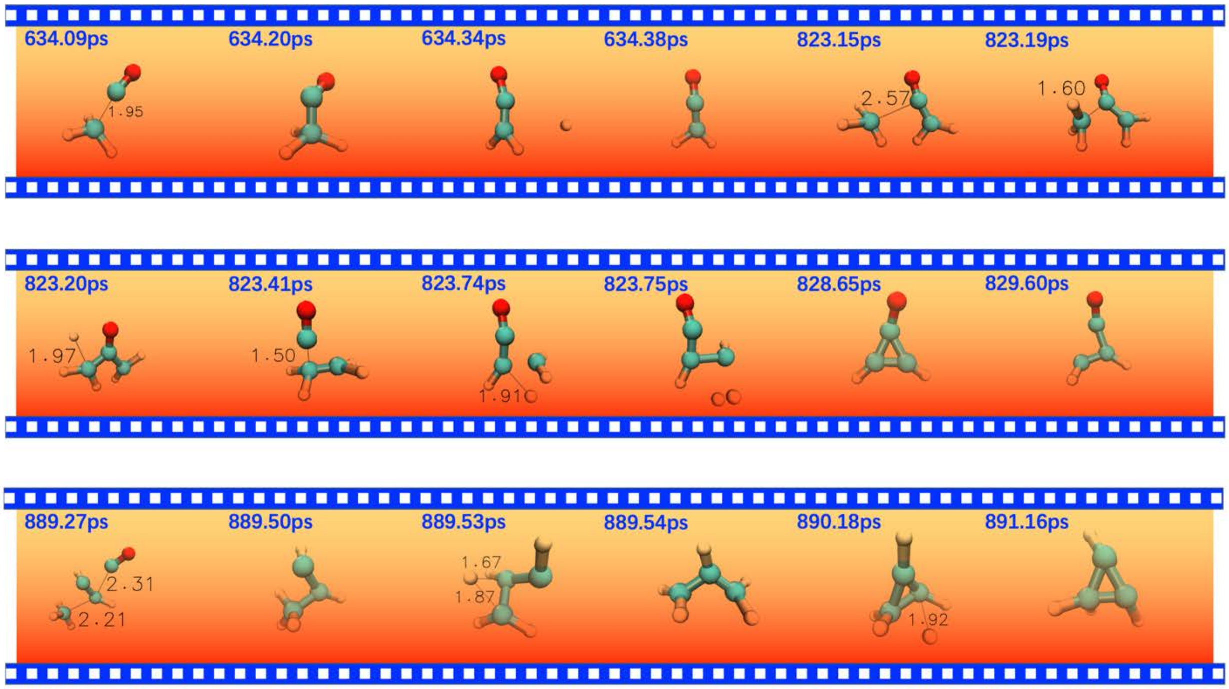

Figure 5. Real-time trajectory of the formation of cyclopropene.

Table 1. Energy prediction errors for the reference set. The mean absolute errors (MAEs) and root mean squared errors (RMSEs) are in eV/atom.

Training Set / Test Set		
Structure Number	MAE	RMSE
578,731 / 13,315	0.041 / 0.140	0.073 / 0.240

Table 2. Rate constants [(cm^3/mol)·s^{-1}] of several reactions detected in the reactive MD simulation with neural network potential and comparison with that in the GRI-Mech 3.0 mechanism.

Reaction	k(exp)[a]	k(simulation)[b]
CH_4 + ·OH → ·CH_3 + H_2O	2.17×10^{13}	1.22×10^{14}
·CH_3 + H_2O → CH_4 + ·OH	4.56×10^{11}	1.49×10^{13}
CH_4 + ·H → ·CH_3 + H_2	4.60×10^{13}	4.34×10^{14}
·CH_3 + H_2 → CH_4 + ·H	2.92×10^{12}	8.49×10^{14}
·CH_3 + HOO· → O_2 + CH_4	1.00×10^{12}	9.39×10^{13}
·H + H_2O → ·OH + H_2	7.17×10^{12}	6.57×10^{12}
CH_4 + HOO· → ·CH_3 + HOOH	1.89×10^{12}	4.79×10^{12}
·O + CH_4 → CH_3 + ·OH	3.96×10^{13}	8.05×10^{13}
O_2 + HOCO → ·CHO + HOO·	1.22×10^{11}	2.91×10^{12}
HOCO + ·OH → ·CHO + H_2O	4.69×10^{13}	1.11×10^{14}

[a]The rate constants were calculated by the Arrhenius equation $k = AT^b e^{\frac{-E_a}{RT}}$, parameters are taken from Ref. 48, and T was set to 3000K.
[b]The rate constants were calculated by the method developed in Ref. 50 and Ref. 51.

Supplementary Note

List of reactions found in the trajectory

[HH]+[H]C([H])[H]->[H]C([H])([H])[H]
[HH]+[H]O->[H]O[H]
[HH]+[H]C[H]->[H]C([H])[H]
O=O->O+O
[H]C([H])=O->[HH]+[H]C=O
OO+[HH]->[H]OO
O+O->OO
[H]OC([H])([H])[H]->[HH]+[H]OC([H])[H]
O=O+[HH]->[H]OO
[H][H]->[HH]+[HH]
O=C=O->C=O+O
[H]C=O->C=O+[HH]
[H]C([H])([H])[H]+[H]O->[H]C([H])[H]+[H]O[H]
[HH]+[H]CO->[H]C([H])=O
[H]C([H])[H]+[H]O->[H]OC([H])([H])[H]
[H]C([H])O->[HH]+[H]C=O
[H]CO->C=O+[HH]
[HH]+[H]C([H])([H])[H]->[H]C([H])[H]+[H][H]
O+[HH]->[H]O
[HH]+[H]C([H])O->[H]C([H])([H])O
O+[H]O[H]->[H]O+[H]O
[H]C([H])([H])O->[HH]+[H]C([H])=O
C=O+O->O=CO
[H]OO[H]->[H]O+[H]O
[HH]+[H]CO->[H]C([H])O
O=O+[H]C([H])([H])[H]->[H]C([H])[H]+[H]OO
[H]C([H])[H]+[H]OO->OO+[H]C([H])([H])[H]
[H]O+[H][H]->[HH]+[H]O[H]
[H]O+[H]OO->O=O+[H]O[H]
[H]C([H])[H]+[H]O->[H]C[H]+[H]O[H]
[H]C([H])([H])[H]+[H]OO->[H]C([H])[H]+[H]OO[H]
OO+[H]O[H]->[H]O+[H]OO
[H]OC([H])([H])[H]->[HH]+[H]C([H])([H])O
[H]C([H])=O+[H]O->[H]C=O+[H]O[H]
OO+[H]C([H])=O->[H]C=O+[H]OO
[HH]+[H]OO->O+[H]O[H]
[H]OO+[H]O[H]->[H]O+[H]OO[H]
O+[H]C([H])([H])[H]->[H]C([H])[H]+[H]O
[HH]+[H]OO->O=O+[H][H]
O=O+[H]C([H])=O->[H]C=O+[H]OO
[H]C([H])=O+[H]O->[H]CO+[H]O[H]
OO+[H][H]->[HH]+[H]OO
[HH]+[H]C([H])[H]->[H]C[H]+[H][H]

CO+O->O=C=O
[H]OC([H])[H]->[HH]+[H]C([H])=O
[H]C([H])=O+[H]C([H])[H]->[H]C([H])([H])[H]+[H]C=O
[H]C([H])([H])[H]+[H]C[H]->[H]C([H])[H]+[H]C([H])[H]
[H]C([H])=O+[H]C([H])[H]->[H]C([H])([H])[H]+[H]CO
[H]OO+[H][H]->[HH]+[H]OO[H]
O=O+[H]C([H])=O->[H]CO+[H]OO
[H]C=O+[H]OO->OO+[H]C([H])O
OO+[HH]->[H]O=O
[H]CO+[H]OO->OO+[H]C([H])=O
[H]OO+[H]OO->OO+[H]OO[H]
[H]OC([H])[H]->[HH]+[H]C([H])O
[H]O+[H]OC([H])([H])[H]->[H]C([H])([H])O+[H]O[H]
[H]CO+[H]OO[H]->[H]C([H])=O+[H]OO
[HH]+[H]OO[H]->[H]O+[H]O[H]
[H]OC([H])([H])[H]->[H]C[H]+[H]O[H]
OO+[H]OC([H])[H]->[H]C([H])=O+[H]OO
[H]O+[H]OC([H])([H])[H]->[H]OC([H])[H]+[H]O[H]
[H]C([H])[H]+[H]OC([H])([H])[H]->[H]C([H])([H])O+[H]C([H])([H])[H]
[H]C([H])C=O->C=O+[H]C[H]
[HH]+[H]OC([H])([H])[H]->[H]OC([H])[H]+[H][H]
[H]OO+[H]OO->O=O+[H]OO[H]
O+[H][H]->[HH]+[H]O
[HH]+[H]C([H])=O->[H]CO+[H][H]
O+[H]C([H])([H])O->[H]C([H])O+[H]O
[HH]+[H]C([H])O->[H]C=O+[H][H]
[H]C([H])=O+[H]OO->[H]C=O+[H]OO[H]
OO+[H]C=O->C=O+[H]OO
[H]C[H]+[H]O[H]->[HH]+[H]OC([H])[H]
O+[H]OO->OO+[H]O
[H]C([H])O+[H]O->[H]C=O+[H]O[H]
[H]C([H])([H])O+[H]O->[H]C([H])=O+[H]O[H]
[H]O+[H]OC([H])[H]->[H]C([H])=O+[H]O[H]
[HH]+[H]C([H])=O->[H]C=O+[H][H]
[H]C(=O)C([H])[H]->[HH]+[H]C([H])C=O
C=O+[H]C[H]->[H]C([H])=C=O
[H]C([H])[H]+[H]OC([H])([H])[H]->[H]C([H])([H])[H]+[H]OC([H])[H]
[H]C([H])O+[H]C([H])[H]->[H]C([H])([H])[H]+[H]CO
OO+[H]C([H])O->[H]CO+[H]OO
[H]CO+[H]OO->O=O+[H]C([H])O
[H]C([H])O+[H]O->[H]CO+[H]O[H]
O+[H]OC([H])([H])[H]->[H]O+[H]OC([H])[H]
[H]C([H])O+[H]C([H])[H]->[H]C([H])([H])[H]+[H]C=O
O=O+[H]OC([H])[H]->[H]C([H])O+[H]OO
OO+[H]CO->C=O+[H]OO
[H]C=O+[H]OO->O=O+[H]C([H])O

[H]C([H])([H])O+[H]O->[H]C([H])O+[H]O[H]
O=O+[H]C=O->C=O+[H]OO
[H]C([H])CO->C=O+[H]C[H]
[HH]+[H]C=O->C=O+[H][H]
[H]O+[H]OC([H])[H]->[H]C([H])O+[H]O[H]
O+[H]C([H])=O->[H]C=O+[H]O
O=O+[H]O->O+[H]OO
O=O+[H]C([H])([H])O->[H]C([H])O+[H]OO
[HH]+[H]OC([H])[H]->[H]C([H])=O+[H][H]
O+[H]C[H]->[HH]+[H]CO
O+[H]C([H])([H])O->[H]C([H])=O+[H]O
O+[H]C([H])[H]->[HH]+[H]C([H])O
OO+[H]C([H])([H])O->[H]C([H])O+[H]OO
OO+[H]C([H])=O->[H]CO+[H]O=O
[H]CO+[H]O=O->OO+[H]C([H])O
[H]C([H])[H]+[H]C([H])[H]->[H]C([H])([H])C([H])([H])[H]
[HH]+[H]C([H])([H])O->[H]C([H])O+[H][H]
C=O+[H]C([H])[H]->[HH]+[H]C([H])C=O
O+[H]C([H])=O->[H]CO+[H]O
O=O+[H]C([H])([H])O->[H]C([H])=O+[H]OO
C=O+[H]C([H])([H])[H]->[H]C([H])[H]+[H]C=O
O+[H]O->O=O+[HH]
OO+[H]OC([H])[H]->[H]C([H])O+[H]OO
[HH]+[H]C=O->O+[H]C[H]
[HH]+[H]C([H])=C=O->[H]C(=O)C([H])[H]
[H]C(=O)C([H])[H]->[HH]+[H]C([H])CO
[H]C([H])([H])=O->[HH]+[H]C([H])=O
[H]C([H])([H])=O->[HH]+[H]C([H])O
CO+O->O=CO
C=O+[H]C([H])([H])[H]->[H]C([H])[H]+[H]CO
C=O+[H]C[H]->[H]C([H])#CO
C=O+[H]O->O=CO+[HH]
OCO->C=O+O
[HH]+[H]CC([H])[H]->[H]C([H])C([H])[H]
[H]C([H])[H]+[H]O->[H]O=C([H])([H])[H]
[H]C=C=O+[H][H]->[HH]+[H]C([H])=C=O
C=O+[H]C->[H]CCO
[H]C=C=O->C=O+[H]C
C=O+[H]O[H]->[H]C=O+[H]O
C=O+[H]OO->O=O+[H]CO
[H]C([H])[H]+[H]O->[HH]+[H]OC([H])[H]
[H]OO->O+[H]O
[H]C([H])([H])[H]->[HH]+[HH]+[H]C[H]
C=O->C+O
[HH]+[H]O[H]->[H]O([H])[H]
[HH]+[H]OO->[H]OO[H]

[H]CC([H])C=O->C=O+[H]CC[H]
[H]C([H])([H])OO->[HH]+[H]C([H])OO
C=O+[H]CC[H]->[H]CC([H])=C=O
[HH]+[H]C([H])=C=O->[H]C([H])([H])CO
[HH]+[H]C([H])CO->[H]C([H])([H])CO
[H]OC([H])[H]->[HH]+[H]CO[H]
C=O+C=O->O=CCO
[H]C([H])=C=O->[HH]+[H]C=C=O
[H]OC([H])[H]->[H]C[H]+[H]O
C=O+[H]O->[H]OC=O
C=O+[H]C([H])[H]->[H]C([H])([H])C=O
O+[HH]+[HH]->[H]O[H]
OO+[H]C([H])[H]->[H]C([H])([H])OO
[HH]+[H]C=C=O->[H]C([H])CO
[H]CC([H])CO->C=O+[H]CC[H]
O=C=O+[HH]->[H]OCO
O=O+O=O->OOOO
OOOO->O=O+OO
[H]OO[H]->O+[H]O[H]
[H]C([H])([H])CO->[HH]+[H]C([H])C=O
[HH]+[H]CCO->[H]C([H])CO
[H]C([H])([H])C=O->[HH]+[H]C([H])=C=O
OOO->O+OO
[H]C([H])([H])C=O->[HH]+[H]C([H])CO
C=O+[H]O->[H]OCO
OOO->O+O=O
O+[H]C[H]->[H]C([H])=O
[H]C([H])C=O->[HH]+[H]C=C=O
[H]OC=O->O=C=O+[HH]
[H]C([H])=C=O->[HH]+[H]CCO
[HH]+[H]C([H])C=O->[H]C([H])([H])C=O
C=O+O=O->O=COO
O=C=O+OO->O=C(O)OO
[H]OCO->O=CO+[HH]
C=O+OO->O=COO
[H]C[H]->[HH]+[H]C
[HH]+[H]CC=O->[H]C([H])C=O
O=O+[H]C([H])[H]->[H]C([H])([H])OO
C=O+OO->OCOO
O=C=O+O=O->O=C(O)OO
[H]O+[H]O[H]->[H]O[H]O[H]
[H]C([H])([H])CO->C=O+[H]C([H])[H]
C=O+[H]C#C[H]->[H]CC([H])C=O
[H]O[H]C([H])([H])[H]->[H]C([H])[H]+[H]O[H]
[H]OC=O->O=CO+[HH]
[HH]+[H]CCO->[H]C([H])C=O

[H]C([H])CO->[HH]+[H]CC=O
OCOO->C=O+O=O
O=C(O)O->O+O=C=O
[H]O[H]OO->O=O+[H]O[H]
[H]C([H])=C=O->[HH]+[H]CC=O
OOOO->OO+OO
[H]C([H])([H])OO->O+[H]C([H])([H])O
C=O+C=O->OCCO
[H]CO[H]->[HH]+[H]OC
OO+[H]O[H]->[H]O[H]OO
OO+[H]O->[H]OOO
O=CC=O->C=O+C=O
[H]OC(=O)O->O=CO+[H]O
C=O+[H]C#C[H]->[H]CC([H])=C=O
[HH]+[H]CC([H])C([H])[H]->[H]C([H])C([H])C([H])[H]
[HH]+[HH]+[H]C->[H]C([H])[H]
[H]C(=O)O->O=CO+[HH]
[H]C([H])([H])[H]+[H]O->[H]O[H]C([H])([H])[H]
[H]C([H])[H]+[H]OO->[H]OOC([H])([H])[H]
[H]OC([H])([H])O[H]->[H]O+[H]OC([H])[H]
O=O+[H]C([H])([H])[H]->[H]C([H])([H])[H]OO
[H]C([H])O->O+[H]C[H]
O=C(O)OO->O=CO+OO
[H]C([H])=C=O->[HH]+[H]C#CO
C=O+[H]C=C[H]->[H]CC([H])C=O
[H]O+[H]OC([H])[H]->[H]CO[H]+[H]O[H]
O=O+[H]C([H])[H]->[H]C[H]+[H]OO
O=C(O)O->O+O=CO
[H]OOO->O=O+[H]O
[H]OC([H])=O->[HH]+[H]OC=O
[H]C([H])([H])[H]->[HH]+[HH]+[HH]+[H]C
[H]OOC([H])([H])[H]->[H]C([H])([H])O+[H]O
[H]OC([H])([H])[H]+[H]OO->[H]OC([H])[H]+[H]OO[H]
[HH]+[H]O[H]->[H][H]O[H]
O=O+[H]OC([H])[H]->[H]CO[H]+[H]OO
[H]OC(=O)O->O=C=O+[H]O
C=O+[H]C([H])C([H])[H]->[H]C([H])C([H])([H])C=O
[H]C=C([H])C=O->C=O+[H]CC[H]
C=O+O=C=O->O=COCO
[H]C([H])C([H])C([H])[H]->[HH]+[H]C([H])CC([H])[H]
[H]C1C([H])([H])C1([H])[H]->[HH]+[H]CC([H])C([H])[H]
[H]C([H])([H])[H]OO->OO+[H]C([H])([H])[H]
O=CO+[HH]->[H]C(O)O
O1OO1->O+OO
[H]C[H]+[H][H]->[H]C([H])([H])[H]
[H]C1([H])[H][H]1->[HH]+[H]C([H])[H]

O=CO+O=O->O=C(O)OO
[H]C(=O)O->O=C=O+[HH]
OO[H]OO->O=O+[H]OO
[H]C([H])C(=O)C([H])([H])[H]->[H]C([H])C=O+[H]C([H])[H]
O=C(O)OCO->O=C=O+O=C=O
[H]OC([H])([H])O->[HH]+[H]OC([H])O
[H]OC([H])([H])O->[HH]+[H]OC([H])=O
[H]C([H])[H]+[H]O[H]->[HH]+[H]OC([H])([H])[H]
C=O+[HH]->[H]OC
[H]CC([H])CO->C=O+[H]C#C[H]
[H]CC([H])([H])C=O->[HH]+[H]CC([H])CO
[HH]+[H]C1C([H])C1([H])[H]->[H]C1C([H])([H])C1([H])[H]
O=C=C(C=O)C=O+[H]O->[H]OC(=O)C(=C=O)C=O
[H]C([H])OO->O+[H]C([H])=O
[H]C([H])(O)O->[HH]+[H]C(=O)O
O+O=O->O1OO1
[H]OO[H]->O+[HH]+[H]O
[H]C[H]+[H]O->[H]C([H])([H])O
OCOO->O+O=C=O
[H][H]C([H])([H])[H]->[HH]+[H]C([H])([H])[H]
OO+[H]OO->[H]OOOO
[H]OOOO->O=O+[H]OO
[H]C[H]+[H]OO->OO+[H]C([H])[H]
[H]OOC([H])([H])[H]->[HH]+[H]OOC([H])[H]
[H]1[H]O1->[HH]+[H]O
O+O+[HH]->[H]OO
O+O=CO->O=COO
O+O=CO->OC(O)O
O=C=O+O=O->OOC(O)O
[H]C([H])CO+[H]C([H])[H]->[H]C([H])([H])C([H])([H])CO
O=C=O+O=CO->O=C(O)OCO
[H]C([H])C(O)C([H])([H])[H]->[H]C([H])C=O+[H]C([H])[H]
[H]OOC(=O)O->O=CO+[H]OO
C=O+O=CO->OCOCO
[H]CO[H]+[H]OO->OO+[H]OC([H])[H]
[H]O[H]O->[H]O+[H]O
[H]C([H])([H])[H]+[H]O->[HH]+[H]OC([H])([H])[H]
[H]O[H]O->O+[H]O[H]
[H]OC([H])([H])CO->[HH]+[H]OC([H])=C=O
OCOCO->C=O+O=C=O
[H]C([H])CO+[H]O->[H]OC([H])([H])CO
[HH]+[H]C#CO->[H]C([H])C=O
[H]OC([H])([H])CO->[H]C([H])=C=O+[H]O
C=O+[H]C([H])C([H])[H]->[H]C([H])C([H])([H])CO
[H]C([H])C([H])([H])CO->[HH]+[H]C([H])C([H])CO
[H]C1([H])C(=O)C1([H])[H]->C=O+[H]C([H])C([H])[H]

[H]C1([H])C(=O)C1([H])[H]->[HH]+[H]C1C(=O)C1([H])[H]
O=CO+[H][H]->[HH]+[H]C(=O)O
[H]CC([H])=C=O->C=O+[H]C=C[H]
C=O+[H]CC([H])[H]->[H]CC([H])([H])C=O
[HH]+[H]CC([H])CO->C=O+[H]CC([H])[H]
C=O+[H]CC([H])[H]->[H]C(=C=O)C([H])[H]
[H]C([H])C([H])C([H])[H]->[HH]+[H]C1C([H])C1([H])[H]
[H]CCC([H])[H]->[HH]+[H]CCC[H]
[H]OC(=O)C(C=O)CO->O=CC(C=O)CO+[H]O
[H]OC([H])([H])O[H]->[HH]+[H]OC([H])O[H]
O=COO->O+O=C=O
OO+[H]CO[H]->[H]CO+[H]OO
[H]C([H])=O->C=O+[HH]+[HH]
[H]C([H])([H])[H]+[H]OO->O+[H]C([H])[H]+[H]O[H]
O=CO=O->C=O+OO
[H]OC([H])([H])[H]->O+[H]C([H])([H])[H]
O+[H]C([H])([H])[H]->[HH]+[H]C([H])([H])O
[H]C([H])[H]+[H]OC([H])[H]->[H]C([H])([H])[H]+[H]CO[H]
OOC1(O)OO1->O=C1OO1+O=O
O=C1OO1+OO->OOC1(O)OO1
OO+[H]OC([H])([H])[H]->[H]C([H])([H])O+[H]OO
OO+[H]C([H])[H]->[H]C([H])([H])O=O
[HH]+[H]OOC([H])[H]->[H]C([H])([H])O+[H]O
[H]C([H])([H])[H]+[H]O->[HH]+[H]C[H]+[H]O[H]
O+[H]O->[H]1OO1
O=CO+OO->OCOOO
[H]C([H])[H]+[H]OO[H]->[H]O+[H]OC([H])([H])[H]
[H]C=O->O+[H]C
[H]C([H])=C=O+[H]C([H])[H]->[H]C([H])C(=O)C([H])([H])[H]
[H]C([H])([H])C([H])([H])CO->[H]C([H])C=O+[H]C([H])[H]
[H]C([H])C(=O)C([H])([H])[H]->[HH]+[H]C([H])C(=O)C([H])[H]
O=O+[H]C(=O)O->O=CO+[H]OO
O+[H]O->[H]O=O
O=O+[H]O([H])[H]->[H]OO+[H]O[H]
O=CO+[H]C([H])[H]->[H]C([H])([H])C(=O)O
OO+[H]OC([H])([H])[H]->[H]OC([H])[H]+[H]OO
OOC(O)O->O=C=O+OO
O+[H]O->O[H]O
[H]O+[H]OC=O->[H]OC(=O)O[H]
OOC(O)O->O=CO+O=O
O+[HH]+[H]C->[H]C([H])=O
[H]C([H])=C=O+[H]C([H])[H]->[H]C([H])([H])C([H])([H])CO
O=C=O+O=CO->O=COC(=O)O
[HH]+[H]OC([H])=C=O->[H]OC([H])([H])C=O
[H]OO+[H]O[H]->[H]O+[H]O+[H]O
[H]C([H])=O+[H]O->[H]OC([H])([H])O

[H]C([H])CO->[HH]+[H]C=CO
O=O+[H]C([H])=O->[H]C([H])(O)OO
O=O+[H]OC([H])([H])[H]->[H]OC([H])[H]+[H]OO
[H]O[H]+[H]O[H]->[H]O+[H]O([H])[H]
OO+[H]CO[H]->[H]OC+[H]OO
[HH]+[H]C1C(O)C1([H])[H]->[H]C1([H])C(=O)C1([H])[H]
[H]C([H])C([H])([H])C=O->[HH]+[H]C([H])C([H])C=O
[HH]+[H]C1C(=O)C1([H])[H]->[H]C([H])C([H])([H])CO
[H]CC([H])CO->C=O+[H]C=C[H]
[HH]+[H]CC([H])=C=O->[H]CC([H])([H])CO
[H]C([H])C([H])C=O->C=O+[H]CC([H])[H]
[H]C1C(O)C1([H])[H]->[HH]+[H]C1C([H])C1O
[H]C([H])C([H])C=O->[HH]+[H]CC([H])=C=O
[H]CC([H])([H])CO->C=O+[H]CC([H])[H]
[H]C([H])C([H])C=O->[HH]+[H]CC([H])CO
[H]CC([H])C(=O)O[H]->[H]CC[H]+[H]OCO
[H]CC[H]+[H]OC=O->[H]CC([H])C(=O)O[H]
[H]C(C=O)C([H])C([H])([H])[H]->C=O+[H]CC([H])C([H])([H])[H]
[H]C1C([H])([H])C1([H])[H]->[HH]+[H]C1([H])CC1([H])[H]
[HH]+[H]C1([H])CC1([H])[H]->[H]C([H])C([H])C([H])[H]
[HH]+[H]CC([H])C([H])[H]->[H]CC([H])([H])C([H])[H]
[H]CC([H])C([H])[H]->[HH]+[H]CC([H])C[H]
[HH]+[H]C1C([H])C1[H]->[H]CC([H])C([H])[H]
[H]C1C([H])C1[H]->[HH]+[H]C1CC1[H]
C=O+OCCCO->OCC(CO)CO
O=C=C(=C=O)=C=O->C=O+OCCCO
O=CC(CO)CO+[H]O->[H]OC(=O)C(=C=O)C=O
[H]OC(=O)C(CO)CO->O=CC(C=O)C=O+[H]O
O=C=C(=C=O)=C=O+[H]O->[H]OC(=O)C(=C=O)=C=O
[H]OC(O)C(=C=O)C=O->O=CC1C(O)C1O+[H]O
O=CC(C=O)CO+[H]O->[H]OC(O)C(C=O)CO
[H]OC(=O)C(=C=O)C=O->O=C=C(=C=O)C=O+[H]O
[H]OC([H])([H])O[H]->[HH]+[H]OC([H])([H])O
[HH]+[H]OC([H])([H])O->[H]OC([H])([H])O[H]2
[H]OC([H])O[H]+[H][H]->[HH]+[H]OC([H])([H])O[H]
OO+[H]O([H])[H]->[H]OO+[H]O[H]
[H]OC1(O)C(=O)C1=C=O->O=CC1C(=O)C1O+[H]O
[H]OC([H])O[H]->[H]CO[H]+[H]O
[H]OCO[H]+[H][H]->[HH]+[H]OC([H])O[H]
O=C=C(C=O)C=O+[H]O->[H]OC(O)C(=C=O)C=O
O=C=C(C=O)C(=O)O->O=CCC=O+O=CO
O=C=CC=O+O=CO->O=C=C(C=O)C(=O)O
O=CC(CO)C(=O)O->O=C=CC=O+O=CO
[H]C([H])([H])OO+[H]C([H])([H])[H]->[H]C([H])[H]+[H]OOC([H])([H])[H]
[H]C([H])([H])OO->[HH]+[H]C1([H])OO1
O+[H]C([H])=O->[H]C([H])(O)O

O+[H]C(=O)O->O=CO+[H]O
[H]OOC([H])([H])[H]->[HH]+[H]C([H])O+[H]O
[H]OO+[H][H]->O+[HH]+[H]O[H]
[H]C([H])[H]+[H]O+[H]O->[H]C([H])([H])[H]+[H]OO
[H]C([H])=O+[H]OC([H])[H]->[H]OC([H])([H])OC([H])[H]
[H]C[H]+[H]O->O+[H]C([H])[H]
[H]O1[H]O1->[H]O+[H]O
[H]CO[H]+[H]OC([H])[H]->[H]OC([H])C([H])([H])O[H]
[H]OO+[H]OO[H]->O=O+[H]O+[H]O[H]
[H]C1([H])([H])[H]O1->[H]C([H])[H]+[H]O
[H]C([H])[H]+[H]OCO->O=CO+[H]C([H])([H])[H]
O+O=C=O->O=C(=O)O
O+OO->O=OO
C=O+OOO->OCOOO
[H]C([H])([H])[H]+[H]OC([H])([H])[H]->[H]C([H])[H]+[H]C([H])[H]+[H]O[H]
[H]C([H])[H]+[H]C([H])[H]+[H]O[H]->[HH]+[H]C([H])([H])[H]+[H]OC([H])[H]
O=O+[H]OO[H]->[H]OO[H]OO
[H]OC([H])[H]+[H]OO->[H]CO[H]+[H]OO[H]
OO+[H]CO[H]->[H]OC([H])OO
[H]OC([H])OO->O+[H]OC([H])=O
[H]OC([H])(O)O->[H]O+[H]OCO
[H]C([H])OO->O+[H]C([H])O
[H]CO+[H]O->[H]OC([H])=O
[H]OC([H])=O->[HH]+[H]C(=O)O
OO+[H]OO[H]->[H]OO[H]OO
[H]OC([H])([H])[H]->[HH]+[H]C[H]+[H]O
[H]C([H])[H]+[H]OC([H])[H]->[H]C([H])([H])[H]+[H]C([H])O
O=C1OO1+O=O->O=OC1(O)OO1
[H]OC([H])[H]->[HH]+[HH]+[H]OC
[H]C([H])([H])[H]+[H]OO->O+[HH]+[H]C[H]+[H]O[H]
O+[H]C[H]+[H]O[H]->[H]C([H])[H]+[H]OO
O=C=O->C+O+O
[H]C([H])OO->[HH]+[H]C(O)O
[HH]+[H]C(O)O->[H]C([H])(O)O
O=O+[H]OC=O->O=C=O+[H]OO
O=O+OO->O=OOO
[H]C([H])([H])OO+[H]C([H])=O->[H]CO+[H]OOC([H])([H])[H]
[H]C([H])([H])OO+[H]C([H])=O->[H]C=O+[H]OOC([H])([H])[H]
OO[H]OO->OO+[H]OO
[H]C([H])[H]+[H]OO->[H]OO[H]C([H])[H]
[H]C([H])([H])[H]+[H]C([H])[H]->[H]C([H])([H])[H]C([H])([H])[H]
OCOOO->O=C=O+OO
O=C=O+[H]OO->O=O+[H]OCO
[H]C([H])=C=O->O+[H]C([H])C
[H]OO+[H]OO->O+OO+[H]O[H]
OO+[H]C([H])[H]->[HH]+[H]C([H])OO

[H]C([H])O+[H]OO[H]->[H]C=O+[H]O+[H]O[H]
O=COO->O+OCO
O+OCO->OC1OO1
[H]C([H])[H]+[H]OO->[H]C[H]+[H]OO[H]
O=O+[H]C([H])O->[H]C([H])(O)OO
O=OC(=O)O->O=C=O+OO
[H]C([H])C(=O)C([H])([H])[H]->[H]C([H])CO+[H]C([H])[H]
[H]C([H])=C=O+[H]C([H])[H]->[H]C([H])([H])C([H])([H])C=O
O+[H]C([H])([H])O->[HH]+[H]C([H])(O)O
O=C=O+[H]OO->[H]OOC(O)O
[HH]+[H]C([H])=C=O->[H]C([H])([H])=C=O
[H]C([H])C=O+[H]C([H])[H]->[H]C([H])=C(O)C([H])([H])[H]
[H]C([H])=C(O)C([H])([H])[H]->[H]C([H])=C=O+[H]C([H])[H]
O=C=O+[H]C([H])([H])[H]->[H]C([H])[H]+[H]OCO
[H]C([H])([H])C(O)O->O=CO+[H]C([H])[H]
OCOO+[HH]->[H]OOC=O
[H]OOCO->O=C=O+[H]O
[H]O+[H]OO->[H]O[H]OO
O=CO+[H]C[H]->[H]C([H])C(=O)O
[H]C([H])(O)C=O->C=O+[H]C([H])=O
O+[H]C([H])([H])[H]+[H]O->[H]C([H])[H]+[H]O[H]O
[H]OC([H])[H]+[H]OO->O+[H]OC([H])([H])O[H]
O+[H]OC([H])([H])O[H]->[H]O+[H]OC([H])([H])O
[H]O+[H]OC([H])([H])O->[H]OC([H])=O+[H]O[H]
O+[H]C([H])([H])O->[H]OC([H])([H])O
[H]OC([H])([H])O->[H]C=O+[H]O[H]
[H]C=O+[H]O[H]->[HH]+[H]OC([H])=O
[H]OC([H])=O->C=O+[H]O[H]
C=O+[H]C=O->[H]C(=O)OC
[H]C(O)OC->C=O+[H]C=O
C=O+[H]C([H])=O->[H]C([H])OCO
[H]OC(O)O[H]->[H]O+[H]OC=O
[H]O+[H]OC=O->O=C=O+[H]O[H]
O=C=O+[H]OO->[H]OOC(=O)=O
[H]C([H])([H])C=O+[H]O->[H]OC(=O)C([H])([H])[H]
[H]OC(=O)C([H])([H])[H]->[H]C([H])[H]+[H]OC=O
C=O+OO->O=OCO
[H]OOOO->OOO+[H]O
C=O+[H]C([H])([H])O->[H]C([H])=O+[H]CO
[H]CO+[H]O[H]->[H]C(O)O([H])[H]
[H]C(=O)O([H])[H]->[H]CO+[H]O[H]
[H]O[H]->O+[H][H]
O=C=O+[H]C([H])[H]->[H]C([H])([H])C(=O)O
O=O+[H]O[H]->[H]O([H])OO
OO+[H]OCO->[H]OC(=O)OO
[H]OC(=O)OO->O=O+[H]OCO

OOO+[H]O[H]->[H]O+[H]OOO
[H]OC([H])([H])[H]+[H]OO->[H]C([H])([H])O+[H]OO[H]
OO+[H]OCO->O=CO+[H]OO
[H]C([H])([H])O+[H]OC([H])[H]->[H]C([H])O+[H]OC([H])([H])[H]
[H]C=O+[H]OO->C=O+[H]OO[H]
[H]C=O+[H][H]->[H][H]C([H])=O
[H][H]C([H])=O->[HH]+[H]C([H])=O
OCOO->O+O=CO
C=O+O=CO->O=COC=O
O=COC=O->C=O+O=C=O
O=O+[H]C([H])[H]->[H]C[H]+[H]O=O
C=O+[H]OO->[H]OOCO
[H]OOC=O->C=O+[H]OO
O+[H]C->[H]CO
[H]C([H])[H]+[H]OO->O+[H]OC([H])([H])[H]
O+[HH]+[H]C->[H]C([H])O
[H]C([H])C([H])O->[HH]+[H]C([H])C=O
O=C=O+O=O->OO=C(O)O
OO=C(O)O->O=CO+OO
C=O+[H]C([H])([H])OO->[H]C([H])([H])OOCO
[H]C([H])([H])OOC=O->O=C=O+[H]C([H])([H])O
[H]C([H])([H])C([H])([H])C=O->[H]C([H])C=O+[H]C([H])[H]
OCO+[H][H]->[HH]+[H]C(O)O
[HH]+[H]C(O)O->O=CO+[H][H]
O+O=C=O->OC1OO1
OOC(O)(O)O->O+O1OC12OO2
O+O1OC12OO2->O=C(O)OOO
O=C(O)OOO->O=C1OO1+O=O
OO+[H]O[H]->[H]O([H])OO
[H]C([H])CO+[H]C([H])[H]->[H]C([H])([H])C=O+[H]C[H]
[H]C([H])([H])C=O+[H]C[H]->[H]C([H])([H])C([H])([H])CO
[H]C([H])([H])CO->[HH]+[HH]+[H]C=C=O
[H][H]C([H])[H]->[HH]+[H]C([H])[H]
[H]C1([H])[H]O1->O+[H]C([H])[H]
O+[H]C([H])[H]->[H]C([H])([H])O
[H]OC([H])[H]->O+[HH]+[H]C[H]
[HH]+[H]C+[H]O->[H]OC([H])[H]
[H]C([H])C=O+[H]O->[H]OC([H])([H])CO
[H]OC([H])([H])CO->C=O+[H]OC([H])[H]
C=O+O=CO->O=COCO
[H]O([H])[H]->[HH]+[HH]+[H]O
O=C=O+[H]OO->OO+[H]OCO
[H]C(=O)=O->O=CO+[HH]
OO+[H]C([H])=O->[H]C(O)[H]OO
[H]OOC(=O)O->O=C=O+[H]OO
[H]C([H])[H]+[H][H]->[H][H]C([H])([H])[H]

[H]C([H])[H]+[H]OO->O=O+[H]C[H]+[H][H]
O=C=O+[H]O->[H]OC(O)O
C=O+O=O->COOO
O=O+[H]OC([H])([H])O->[H]O=C([H])O+[H]OO
[H]OC([H])=O->[H]C=O+[H]O
[H]CO+[H]O->[H]OC([H])O
[H]O+[H]OC([H])=O->[H]OC=O+[H]O[H]
O=CO+[H]O[H]->[H]O[H]OCO
[H]O[H]+[H]O[H]->[H]O[H]O([H])[H]
O=CO+[H]O[H]->[H]O([H])C(=O)O
[H]O([H])C(=O)O->O=C=O+[H]O[H]
[H]O+[H]OO->[H]OOO[H]
OO+[H]OC([H])([H])[H]->[H]C([H])([H])O[H]OO
[H]C([H])([H])O[H]OO->[H]C([H])([H])O+[H]OO
[H]C([H])([H])O+[H]OO->O=O+[H]OC([H])([H])[H]
[HH]+[H]C=CO->[H]C([H])=C=O
C=O+[H]O[H]->[H]O[H]CO
[H]C([H])CO+[H]OC([H])[H]->[H]OC([H])([H])C([H])([H])CO
[H]OC([H])([H])C([H])([H])CO->[H]C([H])=C=O+[H]OC([H])[H]
OO+[H]OC([H])[H]->[H]OC([H])([H])OO
[H]OC([H])OO->O=O+[H]CO[H]
OO+[H]C=O->[H]C(O)OO
[H]C(=O)OO->OO+[H]C=O
[HH]+[H]CO->[H]CO[H]
[H]OC([H])([H])C=O->[H]C([H])=C=O+[H]O
[H]C([H])([H])O->[HH]+[HH]+[H]C=O
OO+[H][H]->O+[H]O[H]
OO+[H]C([H])C=O->[H]C=C=O+[H]OO
O=O+[H]OC([H])([H])[H]->[H]C([H])([H])O[H]OO
O=C=O+O=O->OCOOO
C=O+[H]O+[H]O->OCO+[H]O[H]
O=O+[H]OC->[H]OCOO
O=CO+[H]OO->[H]OOC(O)O
[HH]+[H]C#CO->[H]C([H])CO
O=O+[H]C[H]->[H]C([H])OO
[H]C1([H])OO1->[HH]+[H]C(O)O
[H]O([H])O->O+[H]O[H]
[H]OC([H])([H])C=O->C=O+[H]OC([H])[H]
[H]C([H])O+[H]O->[H]OC([H])([H])O
[H]C([H])([H])[H]+[H]C([H])=C(O)C([H])[H]->[H]C([H])[H]+[H]OC(C([H])[H])C([H])[H]
[H]C([H])[H]+[H]OC(C([H])[H])C([H])[H]->[H]C([H])([H])[H]+[H]C([H])C(O)C([H])[H]
[H]C([H])C([H])([H])CO->[HH]+[H]C([H])C([H])C=O
[H]C([H])C([H])([H])C=O->[HH]+[H]C1C(O)C1([H])[H]
[HH]+[H]C([H])C([H])CO->[H]C1([H])C(=O)C1([H])[H]
[HH]+[H]C([H])C([H])C=O->C=O+[H]C([H])C([H])[H]
[H]C([H])C([H])([H])CO->[HH]+[H]CC([H])([H])CO

[H]C([H])C([H])([H])C=O->C=O+[HH]+[H]CC([H])[H]
C=O+[H]C([H])C([H])[H]->[H]C1([H])C(O)C1([H])[H]
[H]C1([H])C(O)C1([H])[H]->[HH]+[H]C1C(O)C1([H])[H]
[HH]+[H]C1C(O)C1([H])[H]->[H]C([H])C([H])([H])CO
[HH]+[H]C1C(=O)C1([H])[H]->C=O+[H]C([H])=C([H])[H]
C=O+[H]C([H])C([H])[H]->[HH]+[H]CC([H])([H])C=O
[H]C([H])C([H])([H])CO->C=O+[HH]+[H]CC([H])[H]
[H]C([H])C([H])([H])CO->[HH]+[H]C(=C=O)C([H])[H]
[HH]+[H]C(=C=O)C([H])[H]->[H]CC([H])=C=O+[H][H]
C=O+[H]O[H]->[H]O+[H]OC
[H]C([H])([H])O+[H]O[H]->[H]C([H])=O+[H]O([H])[H]
[H]C([H])O+[H]O([H])[H]->[H]C([H])([H])O+[H]O[H]
[H]C1C([H])C1O->C=O+[H]C=C[H]
[H]CC([H])=CO->C=O+[H]CC[H]
CO+[H]CC[H]->[H]CC([H])CO
C=O+[H]CC[H]->[H]C1C([H])C1O
C=O+[H]CC[H]->[H]C=C([H])CO
[HH]+[H]C(=O)O->O=C=O+[H][H]
O=O+OC1OO1->OOC1(O)OO1
O=C(O)O+[H]C([H])([H])[H]->[H]C([H])[H]+[H]OC(=O)O
[HH]+[H]CC([H])C=O->C=O+[H]CC([H])[H]
[H]CC([H])([H])CO->[HH]+[H]C1C(=O)C1[H]
C=O+O=C=O->COC(=O)O
[H]O+[H]O[H]->[H]OO([H])[H]
[HH]+[H]C1C(=O)C1[H]->[H]C1C(O)C1([H])[H]
[HH]+[H]C1C([H])C1O->[H]C1C(=O)C1([H])[H]
[HH]+[H]C1C([H])C1O->[H]CC([H])([H])C=O
[H]CC([H])([H])C=O->[H]C+[H]C([H])=C=O
[H]CC([H])([H])CO->[HH]+[H]CC([H])CO
[HH]+[H]CC([H])=C=O->[H]C([H])C([H])CO
C=O+[H]CC([H])[H]->[H]C1C(O)C1([H])[H]
[HH]+[H]CC([H])CO->[H]C(=C=O)C([H])[H]
[H]C([H])C([H])CO->C=O+[H]CC([H])[H]
[HH]+[H]CC([H])=C=O->[H]C(=C=O)C([H])[H]
[H]C([H])C([H])C=O->[HH]+[H]CC([H])C=O
[H]OC(=O)O[H]->[H]O+[H]OCO
[H]C=C([H])C=O->C=O+[H]C=C[H]
[H]C1C([H])C1O->[HH]+[H]C1CC1O
[HH]+[H]C1CC1O->[H]C1=C([H])C1O
[H]C1C(=O)C1[H]->O+[H]C1CC1[H]
[H]C([H])([H])O+[H]OO->[H]C([H])O+[H]O+[H]O
C=O+O=C=O->O=CC(=O)O
O=C=C(=O)O->C=O+O=C=O
[H]CC([H])CO+[H]O->[H]C(=C)C=O+[H]O[H]
[H]C(=C)C=O+[H]O[H]->[H]CC([H])C=O+[H]O
[H]CC([H])C=O+[H]O->[H]CC([H])C(=O)O[H]

[H]CC([H])C(=O)O[H]->[H]C=C[H]+[H]OC=O
[H]CC([H])C(O)O[H]->[H]CC[H]+[H]OC=O
[H]OC1(O)C([H])C1[H]->[H]CC[H]+[H]OC=O
[H]CC[H]+[H]OCO->C=O+[H]CC([H])O[H]
C=O+[H]CC([H])O[H]->[H]CC([H])C(=O)O[H]
[H]OC1(O)C([H])C1[H]->[H]C1C(=O)C1[H]+[H]O
[H]C1C([H])C1O+[H]O->[H]OC1([H])C([H])C1O
[H]C([H])C=O+[H]OO->[H]C([H])CO[H]OO
O=O+[H]C([H])CO[H]OO->OO[H]OO+[H]C([H])CO
[H]OC([H])C([H])C=O->C=O+[H]CC[H]+[H]O
C=O+[H]CC([H])C=O->[H]C(C=O)C([H])C=O
[H]C(=C=O)C([H])C=O->C=O+[H]CC([H])C=O
O=O+[H]C([H])=C=O->[H]C#CO+[H]OO
C=O+[H]CC[H]->[HH]+[H]CCC=O
[HH]+[H]CCC=O->[H]CC([H])C=O
[HH]+[H]CCC=O->[H]CC([H])CO
[H]CC([H])CO->C=O+[H]C+[H]C
C=O+[H]C+[H]C->[H]CC([H])C=O
[H]OCO->OCO+[HH]
[H]CC([H])O->[HH]+[H]CC=O
[HH]+[H]CCO->[H]CC([H])=O
[H]C1C([H])C1O+[H]OO->OO+[H]OC1C([H])C1[H]
[H]CC([H])=O->[HH]+[H]C=C=O
[H]C([H])CO+[H]OO->O=O+[H]C([H])([H])C=O
OO+[H]OC1C([H])C1[H]->[H]C1C(=O)C1[H]+[H]OO
[H]C1C(=O)C1[H]->C=O+[H]CC[H]
C=O+[H]C([H])[H]+[H]CC[H]->[H]C(C=O)C([H])C([H])([H])[H]
[H]CC([H])C([H])([H])[H]->[H]C([H])[H]+[H]CC[H]
[H]C([H])[H]+[H]CC([H])C=O->[H]C(C=O)C([H])C([H])([H])[H]
[H]CC([H])C([H])([H])[H]->[HH]+[H]CC([H])C([H])[H]
C=O+[H]CC([H])C([H])([H])[H]->[H]C(CO)C([H])C([H])([H])[H]
[H]C(CO)C([H])C([H])([H])[H]->[HH]+[H]C([H])C([H])C([H])C=O
[HH]+[H]C([H])C([H])C([H])C=O->[H]C([H])C([H])([H])C([H])C=O
[H]C([H])C([H])([H])C([H])C=O->[H]C(=C=O)C([H])[H]+[H]C[H]
[H]C(=C=O)C([H])[H]+[H]C[H]->C=O+[H]C([H])C([H])C([H])[H]
C=O+[HH]+[H]C([H])CC([H])[H]->[H]C([H])C([H])(C=O)C([H])[H]
[H]C([H])C(C=O)C([H])([H])[H]->C=O+[HH]+[H]C([H])CC([H])[H]
[HH]+[H]C([H])=CC([H])[H]->[H]C([H])CC([H])([H])[H]
[H]C([H])CC([H])([H])[H]->[HH]+[H]C([H])CC([H])[H]
[H]C1C([H])([H])C1([H])[H]->[HH]+[H]C([H])C([H])([H])C
C=O+[HH]+[H]C([H])C([H])([H])C->[H]C(=C=O)C([H])([H])C([H])[H]
[H]C(=C=O)C([H])([H])C([H])[H]->[H]C#CO+[H]C([H])C([H])[H]
[H]C([H])C([H])[H]+[H]C=C=O->[HH]+[H]C1([H])C(CO)C1([H])[H]
[HH]+[H]C1([H])C(CO)C1([H])[H]->[H]C1([H])C([H])([H])C1([H])CO
[H]C([H])C([H])(CO)C([H])[H]->C=O+[HH]+[H]C([H])CC([H])[H]
C=O+[H]C([H])=C([H])C([H])[H]->[HH]+[H]C([H])CC([H])([H])C=O

[HH]+[H]C([H])CC([H])([H])CO->[H]C(=O)C([H])C([H])C([H])[H]
[H]C(=O)C([H])C([H])C([H])[H]->C=O+[H]C([H])C([H])C([H])[H]
C=O+[H]C([H])C([H])C([H])[H]->[H]C([H])C([H])C([H])([H])CO
[H]C([H])C([H])C([H])([H])CO->C=O+[H]C=C([H])[H]+[H]C[H]
[H]CC([H])[H]+[H]C[H]->[H]C1C([H])([H])C1([H])[H]
[H]OOC=O->O=CO+[H]O
[HH]+[H]C1([H])CC1([H])[H]->[H]CC([H])([H])C([H])[H]
[HH]+[H]C([H])CC([H])[H]->[H]C1C([H])([H])C1([H])[H]
[H]C([H])C([H])C([H])[H]->[HH]+[HH]+[H]CCC([H])[H]
[H]CC([H])C([H])[H]->[H]C([H])C+[H]C[H]
[H]C1C([H])([H])C1([H])[H]->[HH]+[HH]+[H]C1CC1([H])[H]
O=O+[H]C1C([H])([H])C1([H])[H]->[H]CC([H])C([H])[H]+[H]OO
[H]CC([H])([H])C([H])[H]->[HH]+[H]C1C([H])C1([H])[H]
[HH]+[H]CC([H])C([H])[H]->[H]CC([H])C[H]+[H][H]
[H]C1C([H])C1[H]->[HH]+[H]CCC[H]
[H]C1CC1([H])[H]->[HH]+[H]C1CC1[H]
[HH]+[H]C1CC1[H]->[H]CCC([H])[H]
O+O=C=O->OC(O)O
[H]CC([H])C[H]->[HH]+[H]CCC[H]
[H]CCC[H]+[H]O[H]->[H]CC([H])C[H]+[H]O
[H]CCC[H]+[H]OO->OO+[H]CC([H])C[H]
C=O+[H]CCC([H])[H]->[HH]+[H]CCC([H])=C=O
[H]C=CC([H])=C=O->[HH]+[H]C#CCC=O
O=O+[H]CCC=C=O->[H]CCC(CO)OO
[H]CCC(CO)OO->OO+[H]CCC=C=O
OO+[H]CCCC=O->[H]C=CC(CO)OO
[H]CC(C=C=O)OO->OO+[H]CCC=C=O
OO+[H]CCC=C=O->[H]C(CCC=O)OO
[H]C(=O)C(O)CCO->CO+[H]C(=O)C1CO1
CO+[H]C(=O)C1CO1->[H]C(O)C1OC1CO
[H]C(O)C1OC1CO->O=CCCO+[H]CO
OC=CCO+[H]C=O->[H]C(O)C1OC1C=O
O=C=O+[H]C(O)C1OC1CO->[H]C(=O)CC(CO)OC(=O)O
[H]C(=O)CC(CO)OC(=O)O->O=C=O+[H]C(O)C1OC1C=O
[H]C(=O)C1OC1CO->OCC1CO1+[H]C=O
C=O+[H]C(O)C1OC1CO->[H]C(=O)C1OC1COOC
C=O+[H]C(O)C1OC1CO->[H]C(OCO)C1OC1CO
[H]C(OCO)C1OC1C=O->C=O+[H]C(=O)C1OC1CO
[H]C(OCO)C1OC1C=O->C=O+[H]C(O)C1OC1C=O
[H]C(=O)C(O)CCO->O=C=CC=O+[H]C=O
O=C=O+O=C=O->O=COC(=O)O
[H]C([H])[H]+[H]O->[HH]+[H]C1([H])[H]O1
[HH]+[H]C1([H])[H]O1->[H]OC([H])([H])[H]
[H]C([H])=O+[H]OO->O+[H]C=O+[H]O[H]
O=CC#CO+[H]C([H])=O->[H]C([H])(O)C(C=O)C=O
C=O+[H]C([H])(O)C(=C=O)C=O->[H]C([H])(OCO)C(=C=O)C=O

[H]C([H])(OCO)C(=C=O)C=O->O=C=CC=O+[H]C([H])OCO
O=C=CC=O+[H]C([H])OCO->C=O+[H]C([H])(O)C(=C=O)=C=O
[H]OC([H])O->[HH]+[H]C(O)O
[H]C([H])(O)C(C=O)CO->[HH]+[H]C(=O)C(C=O)CO
OO+[H]C(=O)C(=C=O)C=O->O=CC(C=O)CO+[H]OO
O=C=C(=C=O)=C=O+[HH]->[H]OCC(C=O)CO
[H]OCC(C=O)CO->O=C=C(C=O)CO+[HH]
O=C=C(C=O)C=O->C=O+OCCCO
OO+[H]OC=O->[H]OC(=O)OO
[H]OC(O)(O)O->O=C1OO1+[H]O
O=C(O)O+[H]C([H])([H])[H]->O=CO+[H]C([H])[H]+[H]O
OO+[HH]+[H]O[H]->[H]O+[H]OO[H]
[H]OC(=O)C(=C=O)C=O->O=C=C(=C=O)=C=O+[H]O
[H]O+[H]O[H]->[HH]+[H]O[H]O
[H]OC(=O)C(C=O)C=O->OCC(CO)CO+[H]O
OCC(CO)CO+[H]O->[H]OC1(O)C(O)C1C=O
[H]OC(=O)C(=C=O)C=O->O=C1C(O)C1CO+[H]O
O=CC(C=O)C=O+[H]O->[H]OC(=O)C(C=O)CO
O=C=C(C=O)CO+[H]O->[H]OC(=O)C(C=O)C=O
[H]OC(O)C(C=O)C=O->O=C=C(C=O)C=O+[H]O
[H]OC(=O)C(=C=O)C=O->O=C=C(=C=O)CO+[H]O
OCC(CO)CO+[H]O->[H]OC(=O)C(CO)CO
[H]OC(=O)C(=C=O)C=O->C=O+[H]OC(=O)CC=O
C=O+[H]OC(=O)CC=O->[H]OC1(O)C(=O)C1C=O
[H]OC1(O)C(O)C1CO->O=C=C(C=O)C=O+[H]O
O=CC(CO)CO+[H]O->[H]OC(=O)C(=C=O)CO
O=C=C(=C=O)C=O+[H]O->[H]OC(O)C(C=O)C=O
O=COC(=O)O->O=CO+O=CO
[H]OC(=O)C(CO)CO->O=C=C(=C=O)C=O+[H]O
[H]OC1(O)C(=O)C1CO->O=CC1C(=O)C1=O+[H]O
O=C1C(O)C1CO+[H]O->[H]OC1(O)C(=O)C1C=O
[H]OC(=O)C(CO)CO->O=CCCO+[H]OC=O
O=CCCO+[H]OC=O->[H]OC(=O)C(C=O)CO
[H]OC(=O)C(=C=O)C=O->O=C1CC1=O+[H]OC=O
O=C1CC1=O+[H]OC=O->[H]OC(=O)C(=C=O)=C=O
OO+[H]OC=O->O=CO+[H]OO
[H]OOC([H])([H])[H]->[H]O+[H]OC([H])[H]
[H]O=CO->O=CO+[HH]
O=C=C(=C=O)C=O->C=O+O=C=CC=O
C=O+OCCCO->O=CC1C(=O)C1O
O=C=C1C(=O)C1=O->C=O+OCCCO
O=CC(CO)CO->C=O+OCCCO
C=O+OCCCO->O=C1C(=O)C1CO
O=CC1C(=O)C1=O->C=O+O=C1CC1=O
C=O+O=C1CC1=O->O=C=C1C(=O)C1=O
O=C=C(=C=O)C=O->C=O+OCCCO

O=CC(CO)CO+[H]O->[H]OC1(O)C(=O)C1C=O
[H]OC(=O)C(C=O)CO->O=CC(CO)CO+[H]O
O=C=C(C=O)CO+[H]O->[H]OC1(O)C(=O)C1C=O
[H]OC(=O)C(C=O)C=O->O=CC(C=O)C=O+[H]O
O=C=C(C=O)C=O+[H]O->[H]OC1(O)C(=C=O)C1O
O=CC(CO)CO->C=O+O=C=C=C=O
C=O+O=C1CC1O->O=CC1C(=O)C1=O
O=CC(CO)CO->C=O+O=CCCO
C=O+O=C1CC1O->O=CC1C(=O)C1O
O=CC1C(=O)C1O->C=O+O=CCCO
C=O+OCCCO->O=CC(C=O)CO
O=CC(C=O)CO->C=O+O=CCCO
C=O+O=CCCO->O=C=C(=C=O)C=O
[H]OC([H])O[H]->[HH]+[H]OC([H])=O
[H]O+[H]OC([H])O[H]->[H]OCO[H]+[H]O[H]
[H]OC1(O)C(=O)C1=C=O->O=C=C(=C=O)C=O+[H]O
O=C=C(C=O)C=O+O=CO->O=CC(C=O)(CO)C(=O)O
O=C(O)C1(CO)C(=O)C1=O->O=C=C1C(=O)C1O+O=C=O
[H]OC(=O)C(=C=O)=C=O->O=C=C(=C=O)C=O+[H]O
OO+[HH]+[HH]->O+[H]O[H]
O=CO+[H]OC1(O)C(=O)C1C=O->[H]OC(=O)C(C=O)(C=O)C(=O)O
[H]OC(=O)C(CO)(CO)C(=O)O->O=CO+[H]OC(=O)C(=C=O)=C=O
O=CO+[H]OC(=O)C(=C=O)=C=O->[H]OC(=O)C(C=O)(CO)C(=O)O
[H]OC(=O)C(CO)(CO)C(=O)O->O=CO+[H]OC(=O)C(=C=O)C=O
[H]OC(=O)C(CO)(CO)C(=O)O->O=CO+[H]OC(=O)C(C=O)CO
[H]OC(O)C(=C=O)C=O->O=C=C(=C=O)C=O+[H]O
[H]OC(=O)C(CO)CO->O=CC(CO)CO+[H]O
O=CC(CO)CO+[H]O->[H]OC(=O)C(=C=O)=C=O
O=O+[H]OC(=O)C(C=O)CO->[H]OC(=O)C(CO)COOO
[H]OC(=O)C(=C=O)COO=O->OO+[H]OC(=O)C(=C=O)C=O
[H]OC(=O)C(=C=O)=C=O->O=C=C(C=O)C=O+[H]O
O=C=C(=C=O)=C=O+[H]O->[H]OC(=O)C(C=O)C=O
O=C=O+[H]O[H]->[H]O[H]OC=O
[H]C([H])[H]+[H]OC(=O)C(=C=O)=C=O->O=C=C(=C=O)C(=O)O+[H]C([H])([H])[H]
O=CC(CO)C(=O)O->O=C=C=C=O+O=CO
O=C=C=C=O+O=CO->O=C=C(C=O)C(=O)O
O=CO+OCCCO->O=C=C(C=O)C(=O)O
O=CCCO+O=CO->O=C(O)C(CO)CO
O=C(O)C(CO)CO->O=C=CC=O+O=CO
O=CO+O=O->O=COOO
O=C=CC=O+O=CO->O=C=C(=C=O)C(=O)O
O=C=C=C=O+O=CO->O=C(O)C(CO)CO
O=C(O)C(CO)CO->O=C=O+OCCCO
O=C=C=C=O+O=CO->O=CC(C=O)C(=O)O
O=CC(C=O)C(=O)O->O=CCC=O+O=CO
O=C=C(C=O)C(=O)O->O=CCCO+O=CO

[H]CO[H]+[H]O[H]->[H]OC([H])([H])O[H]
[H]C([H])(O)OO->OO+[H]C([H])O
C=O+O=O->C=OOO
[H]C[H]+[H]O[H]->[H]O[H]C([H])[H]
O=CCC=O->CCC=O+O
CCCO+O->OCCCO
O=CC(CO)C(=O)O->O=CCCO+O=CO
O=CO+OCCCO->O=CC(CO)C(=O)O
O=CC(CO)C(=O)O->O=CCC=O+O=CO
O=CO+OC#CCO->O=CC(C=O)C(=O)O
O=CC(C=O)C(=O)O->O=CC#CO+O=CO
O=CC#CO+O=CO->O=C=C(=C=O)C(=O)O
O=C=C(=C=O)C(=O)O->O=CCCO+O=CO
OCCCO->C#CO+C=O
C#CO+C=O->O=CCCO
OCCCO->C=O+CC=O
O=C=C(C=O)C(=O)O->O=CO+OC1CC1O
OC1CC1O+OCO->O=C=C(=C=O)C(=O)O
O=C=C(=C=O)C(=O)O->O=CO+OCCCO
